# Changes in electrodermal activity following sympathicotomy in hyperhidrosis patients

**DOI:** 10.3389/fsurg.2024.1358357

**Published:** 2024-03-11

**Authors:** Ai Van Thuy Ho, Eirik Øvensen, Didrik Lilja, Karin Toska, Odd Grenager, Knut Kristiansen, Jarlis Wesche

**Affiliations:** ^1^ The Faculty of Medicine, Institute of Clinical Medicine, University of Oslo, Oslo, Norway; ^2^ Department of Thoracic and Vascular Surgery, Akershus University Hospital, Lørenskog, Norway; ^3^ The Intervention Centre, Oslo University Hospital, Oslo, Norway; ^4^ The Faculty of Medicine, Institute of Basic Medical Sciences, University of Oslo, Oslo, Norway; ^5^ Department of Medical Biochemistry, Oslo University Hospital, Oslo, Norway

**Keywords:** hyperhidrosis, palmar hyperhidrosis, facial blushing, facial hyperhidrosis, electrodermal activity, endoscopic thoracic sympathicotomy, sympathetic activity, compensatory hyperhidrosis

## Abstract

**Objectives:**

The aim of this study was to assess the potential of electrodermal activity (EDA) as a diagnostic tool for preoperative evaluation in hyperhidrosis patients. EDA levels and patterns in different skin areas were investigated before and after endoscopic thoracic sympathicotomy (ETS) and was compared to healthy subjects.

**Methods:**

Thirty-seven patients underwent two days of measurements before and after the operation. Twenty-five (67.5%) of the patients also had a third measurement after six months. Non-invasive EDA measurements, involving skin conductance, were sampled from five different skin areas while patients were at rest in supine and sitting positions or when subjected to stimuli such as deep inspirations, mental challenge, and exposure to a sudden loud sound.

**Results:**

Prior to the operation, hyperhidrosis patients showed higher spontaneous palm EDA variations at rest and stronger responses to stimuli compared to healthy subjects. Patients with facial blushing/hyperhidrosis or combined facial/palmar hyperhidrosis showed minimal spontaneous activity or responses, particularly during mental challenge and sound stimulus. Notably, palm EDA response was abolished shortly following sympathicotomy, although a minor response was observed after six months. Minimal EDA responses were also observed in the back and abdomen postoperatively.

**Conclusion:**

Hyperhidrosis patients showed stronger EDA response to stimuli compared to healthy subjects. Sympathicotomy resulted in the complete elimination of palm EDA responses, gradually returning to a limited extent after six months. These findings suggest that EDA recordings could be utilized in preoperative assessment of hyperhidrosis patients.

## Introduction

Primary hyperhidrosis is a disorder characterized by sweating in excess of physiological requirements for thermoregulation and heat dissipation. This condition is particularly exacerbated by both physiological and mental stimuli (1–3). Hyperhidrosis primarily affects the palms, soles, axillae, or forehead (face) (1, 2, 4, 5) and is equally prevalent in both genders, with an incidence ranging from 0.6 to 1.0% in the general population (1, 6). The disorder can significantly impact the quality of life for affected individuals (1, 2, 5, 7–9).

Several treatment modalities have been attempted with limited success (1, 7). Endoscopic thoracic sympathicotomy (ETS) is considered the treatment of choice for severe palmar hyperhidrosis (7, 10–13) and facial blushing/hyperhidrosis (14–20). ETS yields satisfactory results for palmar hyperhidrosis, with most severe hand sweating cases being effectively resolved (9, 10, 21, 22). However, the ETS procedure is associated with potentially undesirable side effects, particularly compensatory hyperhidrosis (CH) (23, 24). An expert consensus for the surgical treatment of hyperhidrosis has been published (25), and Weksler et al. reported that R2 sympathicotomy is a suitable surgical option for patients suffering from facial blushing or hyperhidrosis, while R3 sympathicotomy is recommended for palmar hyperhidrosis (26).

Hyperhidrosis is thought to be primarily caused by overactivity of the sympathetic nervous system (27–31), and electrodermal activity (EDA) reflects activity of the sympathetic nervous system. We have previously described EDA responses in healthy subjects using a standardized protocol (32). The aim of the present study was to investigate steady-state levels, dynamics, and recurrence rate of EDA in different skin locations in response to minor physiological and mental stimuli before and after sympathicotomy in hyperhidrosis patients. Additionally, we compared the EDA recordings in these patients to EDA recordings in healthy subjects to assess the protocol’s possible suitability for preoperative diagnostic workup in hyperhidrosis patients.

## Materials and methods

### Subjects

A total of thirty-seven patients were included in the study, all referred from primary healthcare to our hospital from all parts of the country. Patients were scheduled for ETS due to facial blushing (seventeen patients), facial hyperhidrosis (six patients), palmar hyperhidrosis (four patients), and combined facial/palmar hyperhidrosis (ten patients). Twenty patients were not included due to reoperations, missing recordings, or Harlequin sign.

The indication for surgery was disabling hyperhidrosis or facial blushing based on the patients’ complaints. All patients underwent anamnestic and clinical examinations by one of three experienced operating surgeons. Seven patients were smokers. Five patients used medications for comorbidities such as hypertension, asthma, and epilepsy, and two for anxiety. Neither had recordings that differed from the rest.

### Surgical technique

**Figure 1:**
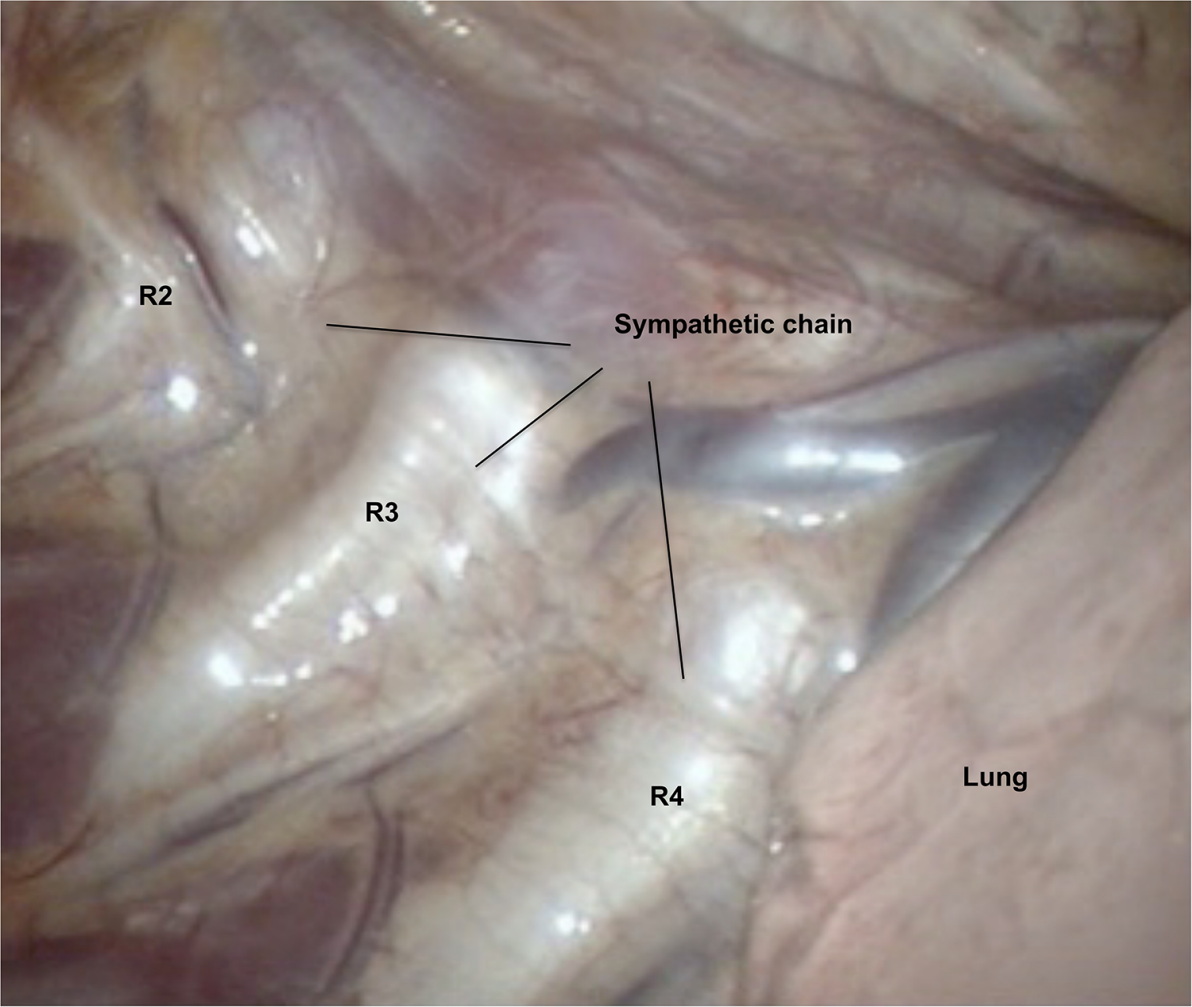
View via videothoracoscope (*R* = rib).

Patients were positioned in a beach-position (back elevated approximately 45 degrees from horizontal), and with abduction of both arms. The surgical procedure was conducted under general anesthesia using single-lumen endotracheal intubation. Video-assisted thoracoscopic surgery was used for all patients. Two 5 mm ports were utilized, with the first positioned in the anterior axillary line in the second to fourth intercostal space, and the second located one to two ribs below the first port. Upon the introduction of thoracoscopy into the thoracic cavity, CO_2_ gas with positive pressure was administered to deflate the lung. Rib-orientation was used to describe the transected position on the sympathetic chain (Figure 1). The costal pleura covering the sympathetic chain was transected using diathermy or ultra-scission scalpel (Ethicon/Johnson & Johnson, New-Brunswik, NJ, USA) at the R2-level (R2 sympathicotomy), R3-level (R3 sympathicotomy) or both R2/R3-level (R2/R3 sympathicotomy). After the insertion of a 10 Fr female urinary catheter into the pleural cavity through the lower port, the lung was reinflated, and the catheter was removed upon cessation of air leakage. If air leakage persisted, the catheter remained in place and was connected either to passive drainage (Heimlich valve) or to a Thopaz pump (Medela, Baar, Switzerland) with active drainage. An identical procedure was then performed on the contralateral side. All patients underwent postoperative chest x-ray before discharge. In the event of a clinically significant pneumothorax or the detection of a large residual gas postoperatively, it was managed with a Tru-close Thoracic Vent (Uresil, Skokie, IL, USA) with passive or active suction, inserted under local anesthesia.

### Experimental design

The present study adopted an identical experimental protocol to that previous described in a study on sweat activity in healthy subjects (32). EDA measurements were obtained continuously over 30 min, with 10 min in a supine position (SUP) and 20 min in a sitting position (SIP). During the last 10 min in the sitting position, the subjects were exposed to a series of stimuli. The first was to take a deep inspiration three times every minute (INSP1, INSP2, INSP3) interspersed with normal respiration between the deep inspirations. This was followed by a mental challenge (MC) in which subjects were required to perform a calculation (subtracting 7 from 100 each time) over the course of one minute. Finally, the subjects were exposed to a sudden (< 0.5 s) sound stimulus (SS). The SS was a single hand clap behind the subject that created a sound of approximately 90 dB.

The subjects wore t-shirt and shorts, and the ambient temperature was maintained at 27 ± 1°C to ensure the subjects were in the thermoneutral zone. They were resting in the supine position for 30 min before each experimental session. All subjects underwent pre- and postoperative measurements. Postoperative measurements were performed four hours to four weeks after ETS. In addition, twenty-five subjects also underwent a third day of measurement six months later.

### Measurements

**Figure 2:**
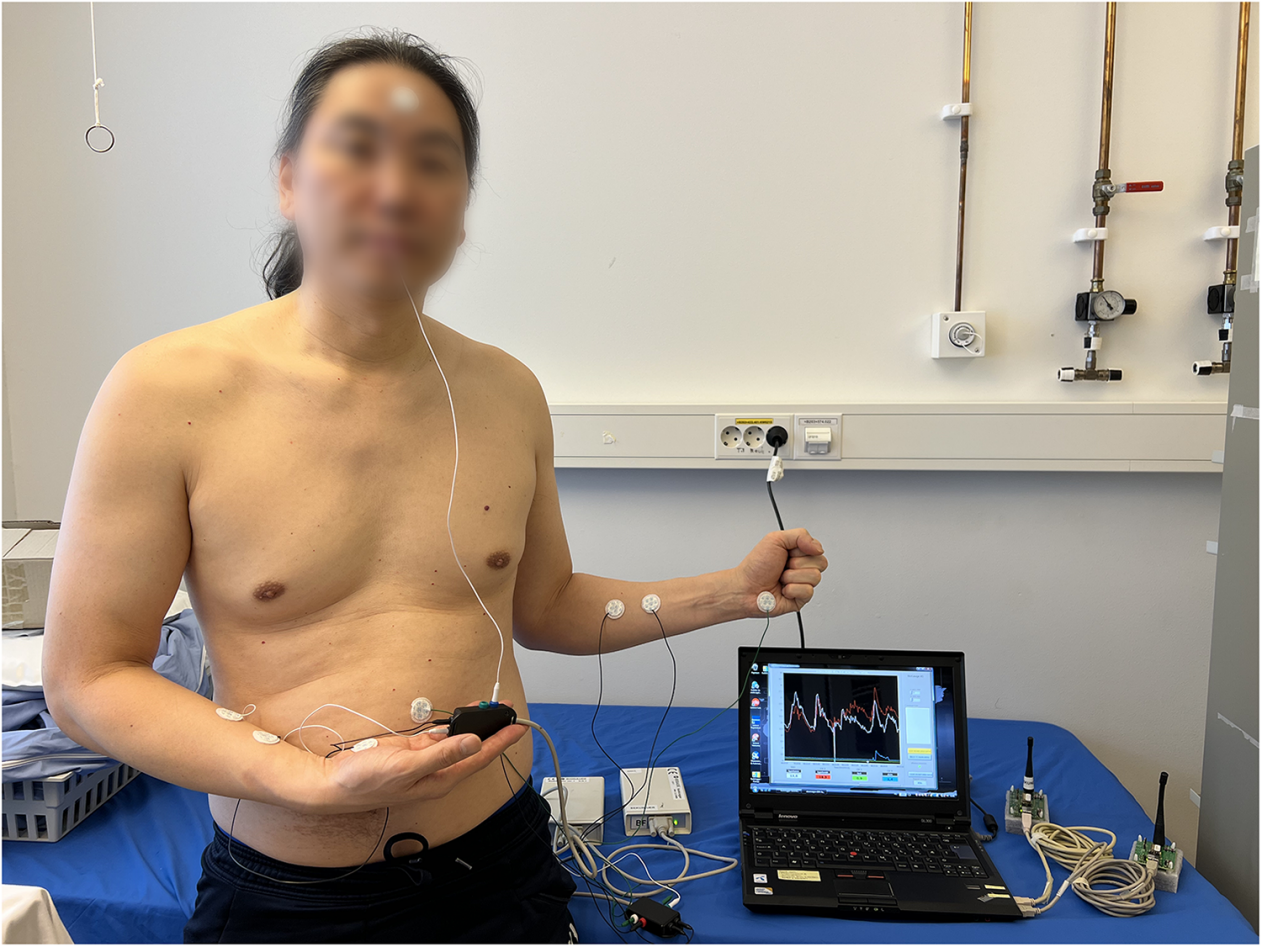
Two Sudologger devices with electrodes attached to a subject. The electrodermal activity was measured in five different skin areas, including the palms, forehead (face), back and abdomen. Additionally, two electrodes on each forearm (reference electrodes) were used for calibrating the measurements of electrodermal activity. The subject has given written consent for publication.

A multichannel Sudologger (BioGauge AS, Oslo Innovation Center, Oslo, Norway) was used to collect non-invasive continuous measurements of electrodermal activity. EDA was simultaneously measured in five locations on both palms, forehead (named face in tables and figures), back, and abdomen (Figure 2) (33). The Sudologger uses skin surface electrodes for unipolar conductance measurements in the stratum corneum. The method was introduced and described in detail by Tronstad et al. (33).

### Data analysis and statistics

Two Sudologger units were used, and recordings of EDA measurements were saved in the Sudologger program. Due to the unavailability of a second instrument, only one Sudologger was used for the first ten patients, and EDA measurements from the left palm, back, face, and abdomen were recorded. Previously, synchronous sweat patterns in both palms have been shown (32, 34), thus measurement from just one palm was considered to be sufficient for further analysis. Data analysis was performed using customized computer programs with Python 2.4 and MATLAB 2021a (MathWork Inc., Natick, MA, USA).

To eliminate random variations, averaged responses from different sweat areas of the skin from thirty-seven patients were calculated (i.e., coherent averaging) (35). Outliers were identified during manual data inspection, including two patients with missing signals over a short period of four to five minutes. The missing signals were attributed to inadequate electrode placement. Instead of removing the entire data points of these outliers, only four to five minutes of missing signal were excluded from the analysis. To ensure consistency in future analysis, these missing data points were replaced with NaN (not a number) values, which would not affect calculation of means or other statistical measures (MATLAB 2021a). This approach allows the remaining data from these patients to contribute to the overall analysis while accounting for the missing information appropriately.

Based on our pilot measurements and observations from similar studies of patients undergoing endoscopic thoracic sympathicotomy, we expected to observe a change of at least 80% in EDA in response to physiological and mental stimuli. A sample size of thirty-seven patients with hyperhidrosis would have 80% power to detect a difference in means of 80% with a standard deviation of 1.05 using a two-sided paired sample *t*-test with an α of 0.05.

For all patients, the statistical significance of differences in sweat patterns between pre- and postoperative days was analyzed using paired-samples *t*-tests in IBM SPSS Statistics 29 (Armonk, NY, USA) (36). Values are expressed as means and 95% confidence intervals (CIs), and differences are considered statistically significant at *p *< 0.05.

## Results

The study population included twenty-one males and sixteen females, with a median age of 32.4 years (range 19–61 years). In our study, 46% of the patients (*n *= 17) had facial blushing, and 16% (*n *= 6) had facial hyperhidrosis and underwent sympathetic chain transection at R2. Four patients (11%) with palmar hyperhidrosis had sympathetic nerve interruption at R3. Ten patients (27%) had sympathetic nerve interruption at R2 and/or R3 for both conditions. One patient with facial blushing underwent sympathetic block by clipping, as requested. This patient had similar EDA recordings as the rest of the group. The ETS procedure was completed within a mean of 20 min (range 16–30 min), with most patients being discharged the next morning. A few patients were discharged in the evening after the operation. Five patients (13.5%) experienced mild perioperative complications such as pneumonia, pneumothorax, and a small lung lesion, but all were discharged within 1–2 days.

**Figure 3:**
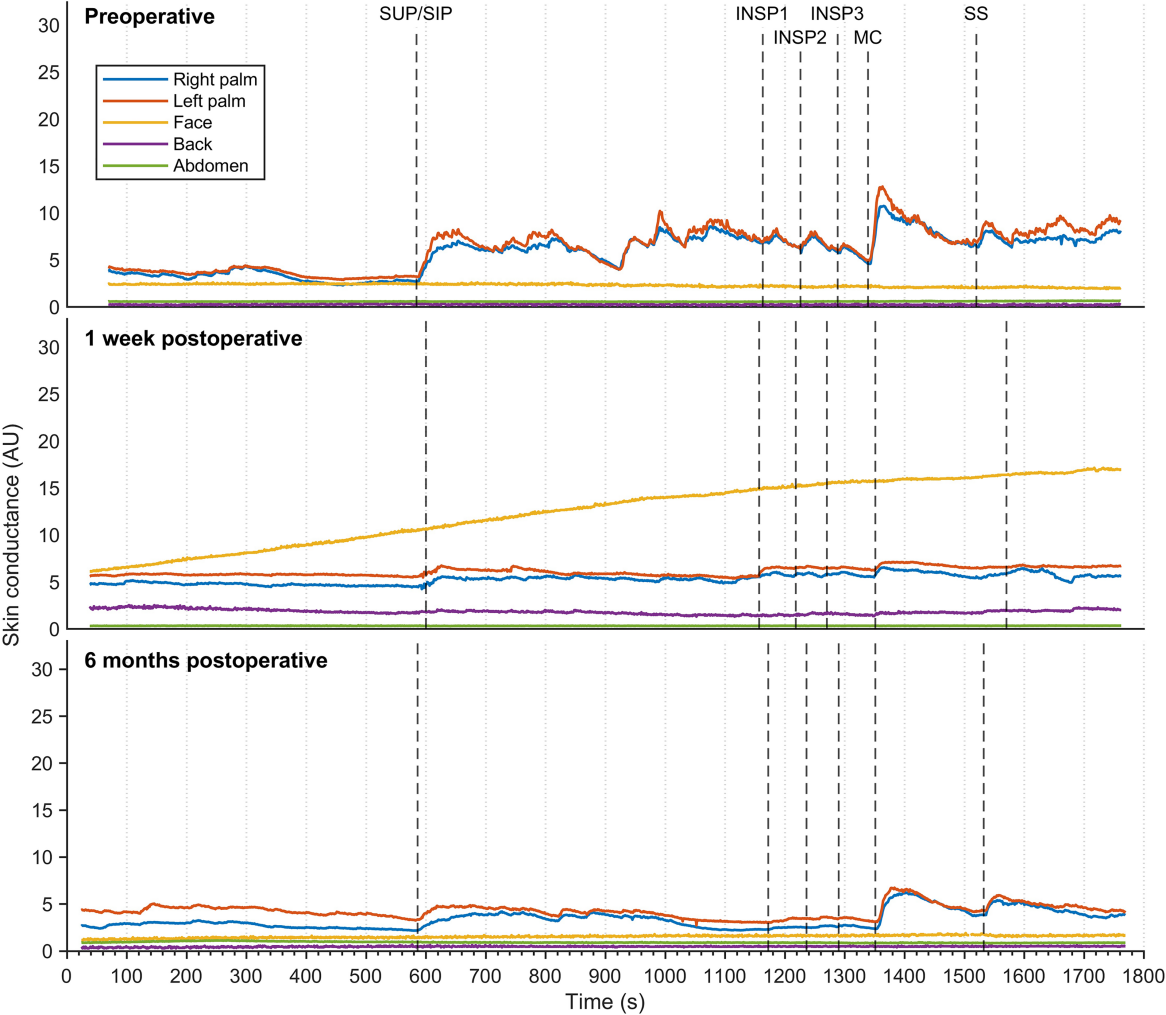
Thirty minutes of continuous electrodermal activity was recorded from five different skin locations in a patient suffering from facial hyperhidrosis, but who was otherwise healthy. Recordings were captured preoperatively (**A**), one week postoperatively (**B**), and six months postoperatively (**C**) The color coding of the traces is explained on the left. The *x*-axis represents time in seconds, while the *y*-axis represents arbitrary units of skin conductance recorded in the right and left hypothenar region of the palm, back, face, and abdomen. Vertical dashed lines indicate the initiation of different stimuli, corresponding to position change from supine (SUP) to sitting (SIP) (SUP-SIP), three deep inspirations (INSP1-3), mental challenge (MC), and sound stimulus (SS).

Figure 3 shows EDA recordings from a patient with facial hyperhidrosis. Preoperatively, the EDA in both left and right palms increased rapidly and synchronously with every stimulus. Additionally, several spontaneous minor electrodermal variations were observed in the palms in the sitting position. However, no EDA responses were observed in the face. The palm responses were completely abolished shortly after the operation, which in this patient was recorded after seven days. However, minor EDA responses were observed in the palms after six months in response to position change, MC, and SS. A gradual increase in facial EDA was observed throughout the experiment after seven days, but there were no measurable responses to individual stimuli. No response recordings to stimuli in EDA were observed from the back and abdominal locations, neither pre- nor postoperatively.

**Figure 4:**
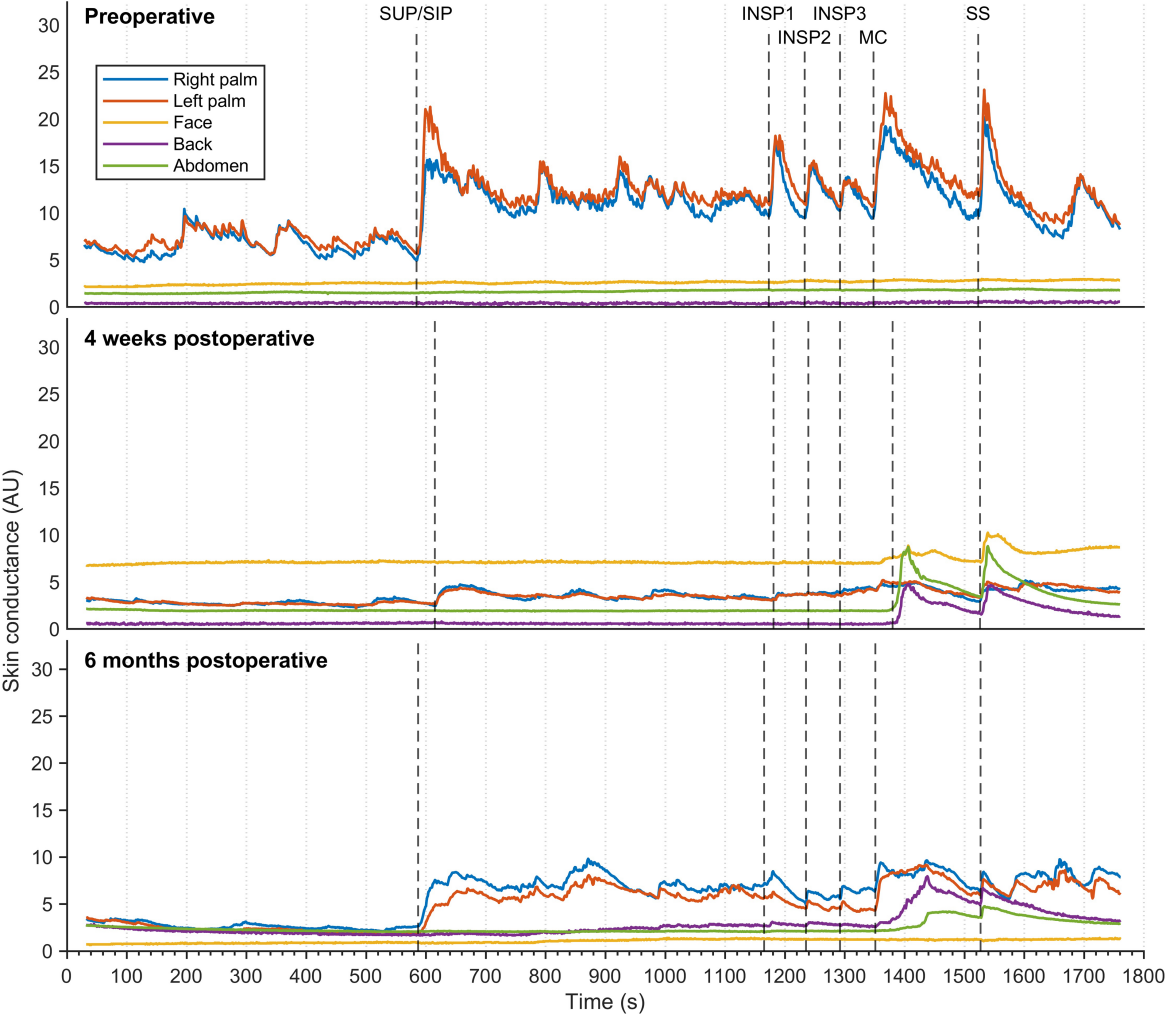
Thirty minutes of continuous EDA was recorded from five different skin areas in a patient suffering from facial blushing, but who was otherwise healthy. Recordings were captured preoperatively (**A**), four weeks postoperatively (**B**), and six months postoperatively (**C**) The color coding, axes, and lines are identical to those in Figure 3.

Another sweat pattern in one patient suffering from facial blushing showed increased palm EDA in response to every stimulus preoperatively (Figure 4), and several small EDA variations were also observed in the supine position. There were no changes in EDA in the face, back, or abdomen. Palm EDA showed lower response to stimuli shortly after ETS (four weeks), but this increased six months postoperatively and was observed in the supine position and in response to every stimulus. However, the amplitude was not as high as it had been preoperatively. Four weeks postoperatively, this patient also presented an EDA response in the face, back, and abdomen upon MC and SS. While the facial EDA response was abolished six months postoperatively, back and abdominal EDA responses to MC and SS stimuli persisted.

**Figure 5:**
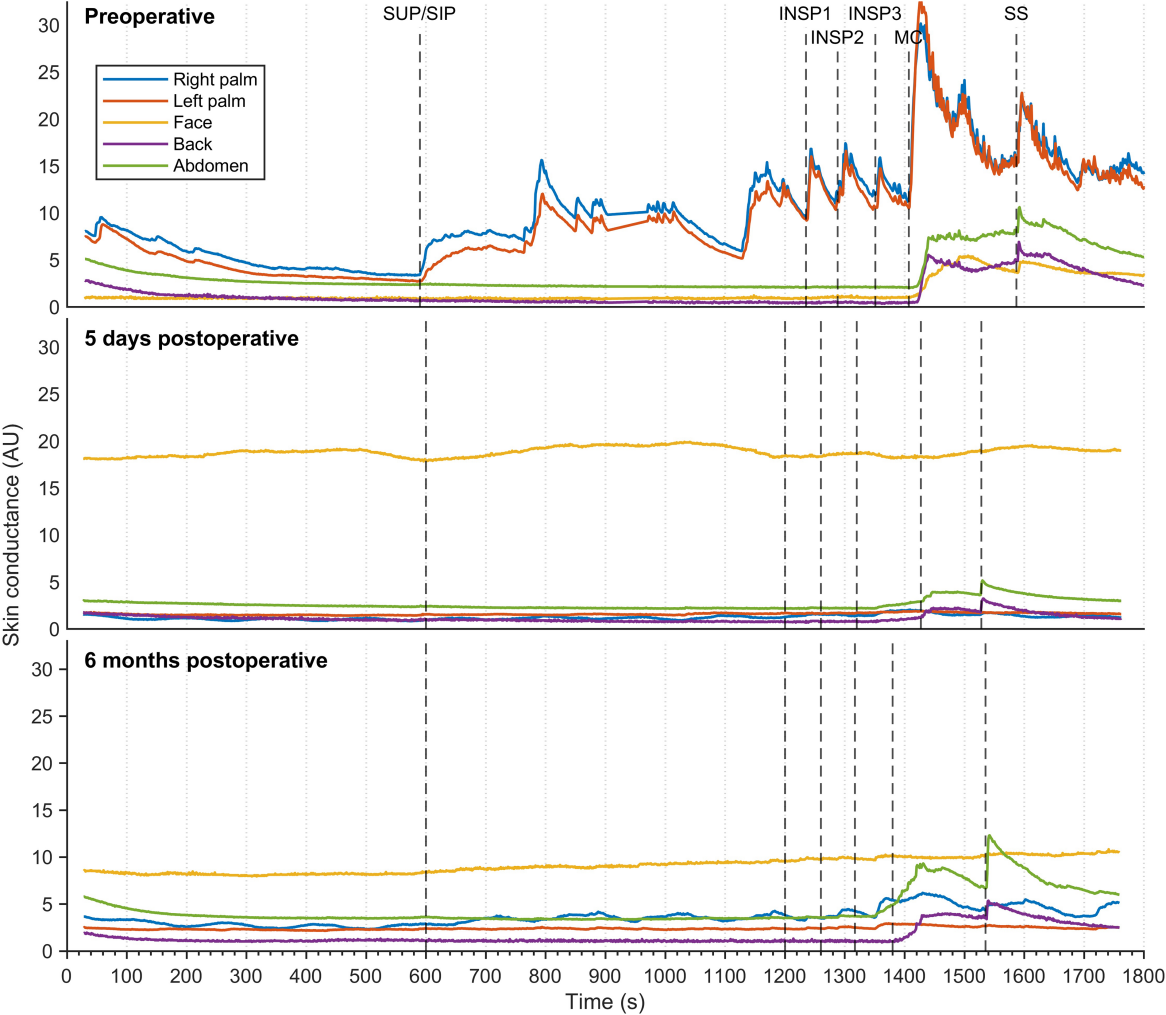
Thirty minutes of continuous EDA was recorded from five different skin areas in a patient suffering from combined palmar/facial hyperhidrosis, but who was otherwise healthy. Recordings were captured preoperatively (**A**), five days postoperatively (**B**), and six months postoperatively (**C**) The color coding, axes, and lines are identical to those in Figure 3.

Figure 5 shows sweat patterns in a patient with combined facial/palmar hyperhidrosis. During preoperative assessment, a slight increase in EDA response was observed in the face, back, and abdomen in response to MC and SS. Shortly after ETS (five days), EDA responses to MC and SS in the back and abdomen were still noticeable and had increased after six months.

**Figure 6:**
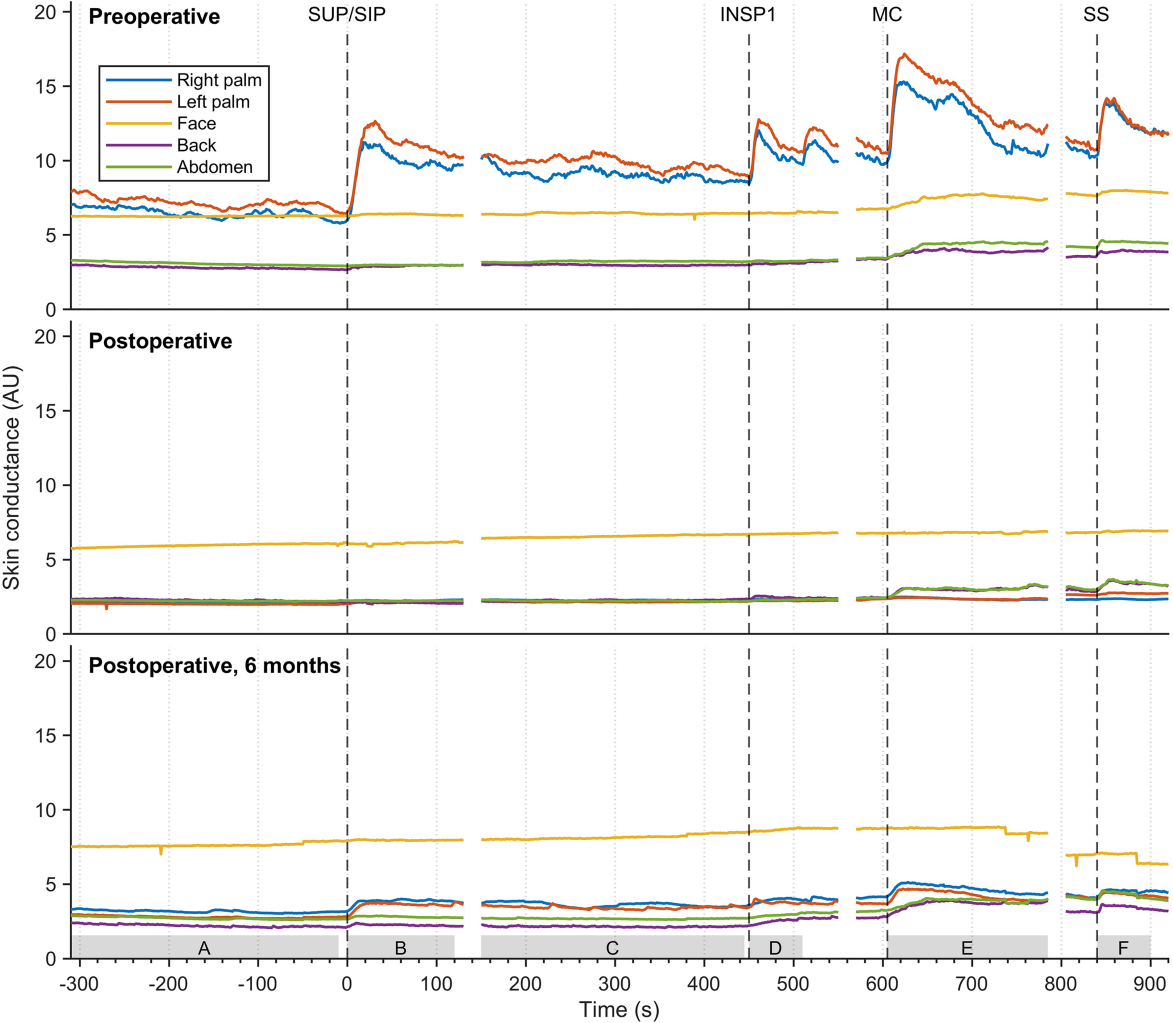
The mean EDA responses of thirty-seven patients with facial blushing/hyperhidrosis, palmar hyperhidrosis, and combined palmar/facial hyperhidrosis preoperatively (**A**), four hours to four weeks postoperatively (**B**), and six months postoperatively (**C**) The color coding, axes, and lines are identical to those in figure 3. The grey boxes on the *x*-axis (A-F) indicate the period from which the values for the different stimuli were calculated in Table 1.

The mean values of continuous EDA recordings from five different skin locations in thirty-seven patients with facial hyperhidrosis, facial blushing, palmar hyperhidrosis, and combined facial/palmar hyperhidrosis are shown in Figure 6. Postoperatively, the EDA pattern differed from preoperative recordings. Before the surgery, EDA peaks varied in magnitude in response to physiological stimuli and mental activity. Recordings from the palms showed a temporary and synchronous response with each stimulus, with the greatest EDA response being recorded during position change, MC, and SS. A slight increase in EDA was also observed in the face, back, and abdomen during MC and upon exposure to SS. Six months postoperatively, an increase in EDA was observed in the palms during position change, MC, and SS, although the amplitude was lower than preoperative recordings. The sweat pattern in the back and abdomen persisted postoperatively, with a somewhat lower EDA amplitude compared to preoperative recordings. After the operation, no EDA response was observed in the face. Supplementary Figure S1 shows the mean EDA response curves, similar to those in Figure 6, for three different groups: patients with facial blushing (seventeen patients), facial hyperhidrosis (six patients), and combined facial/palmar hyperhidrosis (ten patients). The mean recordings in these three groups are very similar.

**Table 1:** Mean values of EDA from five skin areas in hyperhidrosis patients.

Preoperatively hyperhidrosis patients				
SIP_2 min_—SUP_5 min_	Mean	SEM	95% CI	*p*-value (2-tailed)
Right palm	3.67	0.50	2.65, 4.69	<0.001*
Left palm	4.04	0.61	2.80, 5.27	<0.001*
Back	0.13	0.11	−0.10, 0.35	0.127
Face	0.13	0.09	−0.04, 0.31	0.063
Abdomen	−0.08	0.07	−0.22, 0.06	0.128
SIP_5 min_—SUP_5 min_	Mean	SEM	95% CI	*p*-value (2-tailed)
Right palm	2.89	0.50	1.81, 3.96	<0.001*
Left palm	2.86	0.53	1.729, 3.851	<0.001*
Back	0.25	0.20	−0.15, 0.65	0.109
Face	0.20	0.16	−0.11, 0.52	0.101
Abdomen	0.27	0.35	−0.44, 0.97	0.224
INSP1—SIP_5 min_	Mean	SEM	95% CI	*p*-value (2-tailed)
Right palm	1.50	0.41	0.65, 2.34	<0.001*
Left palm	1.48	0.37	0.73, 2.22	<0.001*
Back	0.11	0.14	−0.18, 0.40	0.225
Face	0.04	0.06	−0.09, 0.17	0.280
Abdomen	0.04	0.06	−0.09, 0.17	0.265
MC_3 min_—SIP_5 min_	Mean	SEM	95% CI	*p*-value (2-tailed)
Right palm	4.09	0.50	3.06, 5.12	<0.001*
Left palm	4.53	0.52	3.47, 5.58	<0.001*
Back	0.95	0.23	0.49, 1.42	<0.001*
Face	1.14	0.28	0.57, 1.71	<0.001*
Abdomen	1.13	0.27	0.58, 1.67	<0.001*
SS_1 min_—SIP_5 min_	Mean	SEM	95% CI	*p*-value (2-tailed)
Right palm	3.84	0.55	2.70, 4.99	<0.001*
Left palm	3.75	0.73	2.25, 5.24	<0.001*
Back	1.33	0.36	0.58, 2.08	<0.001*
Face	1.24	0.37	0.48, 2.00	0.001*
Abdomen	1.63	0.40	0.82, 2.44	<0.001*

Postoperatively hyperhidrosis patients				
SIP_2 min_—SUP_5 min_	Mean	SEM	95% CI	*p*-value (2-tailed)
Right palm	0.13	0.06	0.01, 0.25	0.033*
Left palm	0.22	0.07	0.08, 0.35	0.002*
Back	−0.26	0.16	−0.59, 0.08	0.126
Face	0.16	0.08	−0.01, 0.32	0.053
Abdomen	0.01	0.08	−0.14, 0.17	0.857
SIP_5 min_—SUP_5 min_	Mean	SEM	95% CI	*p*-value (2-tailed)
Right palm	0.15	0.05	0.04, 0.25	0.010*
Left palm	0.20	0.05	0.09, 0.31	<0.001*
Back	−0.17	0.18	−0.54, 0.19	0.333
Face	0.68	0.16	0.36, 1.00	<0.001*
Abdomen	0.04	0.12	−0.20, 0.29	0.741
INSP1—SIP_5 min_	Mean	SEM	95% CI	*p*-value (2-tailed)
Right palm	0.10	0.03	0.03, 0.17	0.005*
Left palm	0.07	0.03	0.02, 0.13	0.009*
Back	0.21	0.11	−0.02, 0.43	0.079
Face	0.19	0.07	0.04, 0.33	0.014*
Abdomen	0.04	0.14	−0.24, 0.31	0.786
MC_3 min_—SIP_5 min_	Mean	SEM	95% CI	*p*-value (2-tailed)
Right palm	0.12	0.04	0.03, 0.21	0.014*
Left palm	0.15	0.04	0.06, 0.23	0.002*
Back	0.69	0.23	0.23, 1.15	0.004*
MC_3 min_—SIP_5 min_	Mean	SEM	95% CI	*p*-value (2-tailed)
Face	0.26	0.10	0.06, 0.45	0.012*
Abdomen	0.63	0.23	0.16, 1.10	0.010*
SS_1 min_—SIP_5 min_	Mean	SEM	95% CI	*p*-value (2-tailed)
Right palm	0.17	0.07	0.02, 0.31	0.025*
Left palm	0.15	0.06	0.04, 0.26	0.011*
Back	1.07	0.34	0.36, 1.78	0.005*
Face	0.39	0.17	0.04, 0.73	0.028*
Abdomen	1.00	0.34	0.30, 1.69	0.006*
Six months postoperatively hyperhidrosis patients				
SIP_2 min_—SUP_5 min_	Mean	SEM	95% CI	*p*-value (2-tailed)
Right palm	0.79	0.28	0.20, 1.38	0.012*
Left palm	0.82	0.23	0.33, 1.30	0.002*
Back	0.06	0.17	−0.30, 0.41	0.736
Face	0.23	0.07	0.08, 0.38	0.004*
Abdomen	0.08	0.11	−0.14, 0.30	0.475
SIP_5 min_—SUP_5 min_	Mean	SEM	95% CI	*p*-value (2-tailed)
Right palm	0.88	0.30	0.25, 1.52	0.010*
Left palm	0.65	0.29	0.62, 1.24	0.032*
Back	−0.02	0.24	−0.51, 0.47	0.994
Face	0.60	0.27	0.05, 1.16	0.035*
Abdomen	−0.02	0.24	−0.52, 0.48	0.931
INSP1—SIP_5 min_	Mean	SEM	95% CI	*p*-value (2-tailed)
Right palm	0.41	0.29	−0.20, 1.01	0.173
Left palm	0.32	0.19	−0.06, 0.71	0.093
Back	0.36	0.24	−0.13, 0.84	0.144
Face	0.40	0.15	0.09, 0.71	0.013*
Abdomen	0.22	0.10	0.02, 0.42	0.036*
MC_3 min_—SIP_5 min_	Mean	SEM	95% CI	*p*-value (2-tailed)
Right palm	0.93	0.22	0.45, 1.40	<0.001*
Left palm	0.84	0.19	0.44, 1.24	<0.001*
Back	1.55	0.37	0.79, 2.30	<0.001*
Face	0.54	0.11	0.31, 0.77	<0.001*
Abdomen	1.23	0.32	0.58, 1.89	<0.001*
SS_1 min_—SIP_5 min_	Mean	SEM	95% CI	*p*-value (2-tailed)
Right palm	0.80	0.27	0.22, 1.38	0.010*
Left palm	0.86	0.15	0.54, 1.18	<0.001*
Back	1.52	0.52	0.42, 2.62	0.010*
Face	0.53	0.11	0.29, 0.77	<0.001*
Abdomen	1.62	0.55	0.46, 2.78	0.009*

Preoperatively vs. postoperatively hyperhidrosis patients				
SIP-SUP_2 min_	Mean	SEM	95% CI	*p*-value (2-tailed)
Right palm	3.55	0.49	2.53, 4.56	<0.001*
Left palm	3.82	0.61	2.59, 5.06	<0.001*
Back	0.33	0.20	−0.08, 0.73	0.110
Face	−0.02	0.12	−0.28, 0.23	0.853
Abdomen	−0.09	0.10	−0.31, 0.12	0.376
SIP-SUP_5 min_	Mean	SEM	95% CI	*p*-value (2-tailed)
Right palm	2.75	0.48	1.76, 3.74	<0.001*
Left palm	2.69	0.54	1.59, 3.80	<0.001*
Back	0.42	0.27	−0.12, 0.96	0.119
Face	−0.48	0.25	−0.98, 0.02	0.061
Abdomen	0.23	0.35	−0.49, 0.95	0.529
INSP1	Mean	SEM	95% CI	*p*-value (2-tailed)
Right palm	1.35	0.42	0.49, 2.22	<0.001*
Left palm	1.41	0.37	0.66, 2.15	<0.001*
Back	−0.09	0.18	−0.46, 0.27	0.614
Face	−0.15	0.10	−0.35, 0.06	0.153
Abdomen	0.00	0.16	−0.33, 0.34	0.981
MC	Mean	SEM	95% CI	*p*-value (2-tailed)
Right palm	3.97	0.50	2.94, 5.01	<0.001*
Left palm	4.38	0.53	3.30, 5.47	<0.001*
Back	0.26	0.27	−0.29, 0.81	0.347
Face	0.89	0.32	0.25, 1.52	0.008*
Abdomen	0.50	0.38	−0.27, 1.26	0.200
SS	Mean	SEM	95% CI	*p*-value (2-tailed)
Right palm	3.76	0.56	2.61, 4.91	<0.001*
Left palm	3.60	0.73	2.09, 5.11	<0.001*
Back	0.26	0.40	−0.57, 1.10	0.518
Face	0.86	0.43	−0.02, 1.73	0.055
Abdomen	0.63	0.53	−0.46, 1.72	0.243

Preoperatively vs. six months postoperatively hyperhidrosis patients				
SIP-SUP_2 min_	Mean	SEM	95% CI	*p*-value (2-tailed)
Right palm	2.53	0.64	1.18, 3.90	0.001*
Left palm	3.06	0.58	1.86, 4.25	<0.001*
Back	0.15	0.18	−0.21, 0.52	0.399
Face	−0.04	0.10	−0.25, 0.17	0.709
Abdomen	−0.08	0.14	−0.36, 0.20	0.547
SIP-SUP_5 min_	Mean	SEM	95% CI	*p*-value (2-tailed)
Right palm	2.15	0.57	0.92, 3.37	0.002*
Left palm	2.36	0.56	1.21, 3.52	<0.001*
Back	0.46	0.33	−0.21, 1.14	0.169
Face	−0.51	0.34	−1.21, 0.19	0.143
Abdomen	0.12	0.30	−0.50, 0.74	0.696
INSP1	Mean	SEM	95% CI	*p*-value (2-tailed)
Right palm	1.19	0.52	0.09, 2.29	0.036*
Left palm	1.11	0.43	0.23, 1.99	0.016*
Back	−0.29	0.29	−0.88, 0.30	0.321
Face	−0.29	0.14	−0.58, 0.01	0.057
Abdomen	−0.18	0.12	−0.43, 0.08	0.163
MC	Mean	SEM	95% CI	*p*-value (2-tailed)
Right palm	2.83	0.59	1.58, 4.08	<0.001*
Left palm	3.40	0.68	2.00, 4.79	<0.001*
Back	−0.58	0.38	−1.36, 0.20	0.138
Face	0.42	0.32	−0.25, 1.09	0.211
Abdomen	−0.32	0.28	−0.89, 0.25	0.260
SS	Mean	SEM	95% CI	*p*-value (2-tailed)
Right palm	2.41	0.59	1.15, 3.68	0.001*
Left palm	2.40	0.80	0.69, 4.10	0.009*
Back	−0.25	0.54	−1.39, 0.89	0.646
Face	0.78	0.41	−0.10, 1.65	0.079
Abdomen	0.01	0.45	−0.94, 0.96	0.985
SIP-SUP_2 min_	Mean	SEM	95% CI	*p*-value (2-tailed)
Right palm	0.62	0.21	0.17, 1.06	0.009*
Left palm	0.57	0.19	0.19, 0.96	0.005*
Back	0.25	0.17	−0.11, 0.61	0.161
SIP-SUP_2 min_	Mean	SEM	95% CI	*p*-value (2-tailed)
Face	0.04	0.14	−0.24, 0.32	0.770
Abdomen	0.05	0.13	−0.22, 0.33	0.705
SIP-SUP_5 min_	Mean	SEM	95% CI	*p*-value (2-tailed)
Right palm	0.72	0.26	0.17, 1.27	0.013*
Left palm	0.54	0.25	0.03, 1.05	0.038*
Back	−0.01	0.27	−0.57, 0.56	0.981
Face	−0.20	0.37	−0.96, 0.56	0.594
Abdomen	−0.08	0.18	−0.45, 0.29	0.669
INSP1	Mean	SEM	95% CI	*p*-value (2-tailed)
Right palm	0.30	0.30	−0.33, 0.92	0.329
Left palm	0.22	0.20	−0.19, 0.63	0.287
Back	0.20	0.25	−0.33, 0.72	0.443
Face	0.24	0.17	−0.10, 0.58	0.155
Abdomen	0.15	0.09	−0.04, 0.34	0.124
MC	Mean	SEM	95% CI	*p*-value (2-tailed)
Right palm	0.79	0.21	0.34, 1.24	0.002*
Left palm	0.65	0.18	0.27, 1.04	0.002*
Back	0.97	0.34	0.27, 1.68	0.009*
Face	0.23	0.18	−0.14, 0.60	0.218
Abdomen	0.44	0.35	−0.28, 1.15	0.220
SS	Mean	SEM	95% CI	*p*-value (2-tailed)
Right palm	0.67	0.27	0.09, 1.25	0.025*
Left palm	0.66	0.17	0.30, 1.02	0.001*
Back	0.61	0.46	−0.36, 1.58	0.205
Face	0.00	0.28	−0.59, 0.60	0.996
Abdomen	0.45	0.60	−0.82, 1.72	0.467

Mean values, SEM, and 95% CI for EDA measured from the right and left palm, back, face, and abdomen of thirty-seven hyperhidrosis patients who suffered from facial blushing/hyperhidrosis, palmar hyperhidrosis, and combined palmar/facial hyperhidrosis. The measurements were taken during different stimuli, such as the position change from SUP to SIP (SIP_2 min_—SUP_5 min_, for 2 min; and SIP_5 min_—SUP_5 min_, for 5 min), INSP1 (for 1 min), MC (for 3 min), and SS (for 1 min). The mean values are reported preoperatively, short-term (four hours to four weeks after the operation), and six months postoperatively and as a comparison between these three time points. These mean values were calculated from the periods indicated by the grey boxes marked with (**A–F**) in Figure 6. Supine position values were calculated as the average during a 5 min period (SUP_5 min_) (**A**), while values in the sitting position were calculated 2 min after the position change (SIP_2 min_) (**B**), and 5 min later in the experiment, which represents the baseline values in the sitting position (SIP_5 min_) (**C**). The time period marked with the grey box C represents the baseline for INSP1, MC, and SS. EDA during INSP1 and SS was calculated as the average 1 min after each stimulus, while the MC lasted for 3 min. The values were estimated using paired *t*-test means and 95% confidence intervals for the group of thirty-seven hyperhidrosis patients.

* The asterisk (*) indicates a significant difference from baseline (*p* < 0.05 for a two-tailed test).

SEM, standard error of the mean; CI, confidence interval; EDA, electrodermal activity; SUP, supine position; SIP, sitting position; INSP1, first inspiration; MC, mental challenge; SS, sound stimulus.

Table 1 shows a comparison of postoperative EDA responses to preoperative responses. The table shows a significant difference in postoperative EDA recordings compared to preoperative EDA recordings in the palms (*p* < 0.05) for all thirty-seven patients in response to every stimulus. A significant change after the operation was observed in facial EDA responses to MC. However, no significant differences in EDA were observed between preoperative EDA and shortly postoperative EDA in the face, back, and abdomen in response to the other stimuli (*p* > 0.05). The table also shows that there was a significant increase in amplitude of MC-induced EDA in the back six months after the operation, compared to EDA responses recorded shortly after the operation.

## Discussion

One major finding of this study is the complete abolition of palm EDA responses to stimuli immediately after ETS at the R2-/R3-level, followed by a gradual return of EDA responses at six months. The response was most pronounced during position change, MC, and SS, but with a lower amplitude compared to preoperative recordings. This phenomenon may be attributed to the ongoing activity of EDA responses at the R4-level, as suggested in previous studies (13, 37–48).

Prior to sympathicotomy, the EDA responses were synchronous in both palms, and the responses were comparable to previous findings in healthy subjects, as described by Ho and colleagues (32). However, the patients showed much higher variations in spontaneous palm EDA at rest than the healthy subjects. The responses to stimuli were similar but higher in amplitude. Prior to ETS, there were significant differences in palmar EDA between the hyperhidrosis patients and the thirteen healthy subjects (Table 1) in response to each stimulus. The spontaneous variations in palm EDA during rest in hyperhidrosis patients’ needs further investigation and may be used for diagnostic purposes.

Prior to ETS, minimal EDA responses were observed in the face, back, and abdomen in hyperhidrosis patients, whereas no such EDA responses were seen in healthy subjects.

Only minor spontaneous activity or responses to stimuli were observed in the forehead of patients with facial blushing/hyperhidrosis or combined facial/palmar hyperhidrosis. This observation may be attributed to the method and experimental setup used, as EDA is based on an increase in skin conductance due to sweat secretion, whereas facial blushing and hyperhidrosis may not sufficiently moisten the skin to the same extent as in the palms. Additionally, it is possible that the applied stimuli are not sufficiently strong to elicit EDA responses. Further, a minor response in facial EDA was elicited by MC and SS stimuli in both pre- and postoperative settings in a few patients, as shown in Figure 4. An explanation could be that MC and SS stimuli might elicit a stronger psychological response in facial EDA than exposure to other physiological stimuli.

Recurrence of minor postoperative EDA responses was observed in the back and abdomen in half of the patients, but there were no statistically significant differences in EDA pre- and postoperatively in these locations (Table 1). Previous studies have predominantly relied on patient-reported symptom questionnaires, rather than objective measurements, to evaluate hyperhidrosis and compensatory hyperhidrosis (7, 9, 16, 17, 37, 49–54). Although two different methods for objectively recording sweat have been used in two hyperhidrosis studies, they were limited to 10-second recordings (34, 43).

None of the patients in our study complained of postoperative compensatory hyperhidrosis. However, the present study only lasted six months, and our aim was not to study such phenomena. Long-term studies are needed to address the problem of postoperative compensatory hyperhidrosis.

### Limitations

The study has certain limitations. Firstly, the heterogeneity within the study group, which included individuals with symptoms as blushing and hyperhidrosis in different areas as face, palms, and combinations thereof, represents a challenge. Although presenting results for specific subgroups defined by symptom types and hyperhidrosis locations would have been preferable, the limited sample size of these subgroups prevented statistical evaluation. However, Supplementary Figure S1 shows that there is probably no major difference between the three groups. Secondly, this study was based on EDA recordings that measure sweating elicited by minor stimuli, and not skin blood flow. This might explain why facial blushing did not alter the EDA recordings. Thirdly, despite an identical setup and our efforts to expose hyperhidrosis patients and healthy subjects to the same experimental conditions, the recordings for these groups were not conducted during the same time period. Furthermore, the early postoperative EDA recordings ranged from four hours to four weeks, and thus introduces a variability which represents a limitation regarding the impact of sympathicotomy on EDA patterns in hyperhidrosis patients. The postoperative recordings of Figures 3–5 are very similar, so this is probably not a major limitation to the reliability of the study. The variability was the result of a busy and challenging clinical setting, where patients often sought early discharge due to factors such as long travel distances and high satisfaction with the operation. These limitations emphasize the need for caution in generalizing our findings and highlight opportunities for future research to address these challenges.

## Conclusion

In conclusion, hyperhidrosis patients demonstrated significantly higher amplitudes of palmar EDA responses to stimuli compared to healthy subjects before sympathicotomy. The patients also showed more pronounced variations in palm EDA during rest. Palm EDA responses were completely abolished after ETS but reappeared to some extent at six months. EDA recordings could be useful for preoperative assessment of hyperhidrosis patients and should be explored in future studies.

## Data Availability

The original contributions presented in the study are included in the article/**Supplementary Material**, further inquiries can be directed to the corresponding author.
