# Supplementary material for: Changes in electrodermal activity following sympathicotomy in hyperhidrosis patients
Source: Front Surg. 2024 Mar 11;11:1358357. doi: 10.3389/fsurg.2024.1358357 (PMC10961364; doi:10.3389/fsurg.2024.1358357)
Supplement: Supplementary file 1 [file Table1.docx]

**Preoperatively hyperhidrosis patients vs Day 1 healthy subjects**

| SUP_5min_ | Mean | SEM | 95 % CI | p-value (2-tailed) |
| --- | --- | --- | --- | --- |
| Right palm | 5.39 | 1.25 | 2.82, 7.95 | < 0.001* |
| Left palm | 6.14 | 1.17 | 3.78, 8.50 | < 0.001* |
| Back | 0.79 | 0.86 | -0.94, 2.52 | 0.363 |
| Face | 1.06 | 1.82 | -2.60, 4.72 | 0.563 |
| Abdomen | 1.09 | 0.67 | -0.26, 2.44 | 0.111 |

| SIP_5min_ | Mean | SEM | 95 % CI | p-value (2-tailed) |
| --- | --- | --- | --- | --- |
| Right palm | 5.52 | 1.68 | 2.12, 8.93 | 0.002* |
| Left palm | 6.53 | 1.89 | 2.72, 10.33 | 0.001* |
| Back | 1.06 | 1.02 | -0.99, 3.11 | 0.303 |
| Face | 0.26 | 2.02 | -3.80, 4.31 | 0.898 |
| Abdomen | 1.17 | 0.92 | -0.69, 3.03 | 0.213 |

| INSP1 | Mean | SEM | 95 % CI | p-value (2-tailed) |
| --- | --- | --- | --- | --- |
| Right palm | 5.12 | 2.18 | 0.72, 9.53 | 0.024* |
| Left palm | 6.33 | 2.17 | 1.97, 10.69 | 0.005* |
| Back | 0.82 | 1.10 | -1.40, 3.03 | 0.462 |
| Face | -0.09 | 2.10 | -4.32, 4.14 | 0.967 |
| Abdomen | 1.02 | 0.96 | -0.90, 2.94 | 0.292 |

| MC_3min_ | Mean | SEM | 95 % CI | p-value (2-tailed) |
| --- | --- | --- | --- | --- |
| Right palm | 5.79 | 2.14 | 1.45, 10.14 | 0.010* |
| Left palm | 6.84 | 2.20 | 2.40, 11.27 | 0.003* |
| Back | 1.46 | 1.01 | -0.57, 3.49 | 0.155 |
| Face | 0.55 | 2.22 | -3.91, 5.01 | 0.806 |
| Abdomen | 1.44 | 0.80 | -0.18, 3.06 | 0.081 |

| SS_1min_ | Mean | SEM | 95 % CI | p-value (2-tailed) |
| --- | --- | --- | --- | --- |
| Right palm | 5.66 | 2.14 | 1.33, 9.99 | 0.012* |
| Left palm | 5.40 | 2.22 | 0.91, 9.90 | 0.020* |
| Back | 1.52 | 0.93 | -0.36, 3.41 | 0.111 |
| Face | 1.38 | 2.47 | -3.62, 6.38 | 0.578 |
| Abdomen | 1.55 | 0.85 | -0.18, 3.28 | 0.078 |
