# Supplementary material for: Changes in electrodermal activity following sympathicotomy in hyperhidrosis patients
Source: Front Surg. 2024 Mar 11;11:1358357. doi: 10.3389/fsurg.2024.1358357 (PMC10961364; doi:10.3389/fsurg.2024.1358357)
Supplement: Supplementary file 2 [file Image1.pdf]

FIGURE S1 (supplementary) Shows the mean EDA responses of three groups of patients; facial blushing (seventeen patients) (A), facial hyperhidrosis (six patients) (B), and combined palmar/facial hyperhidrosis (ten patients) (C) preoperatively (upper panel), four hours to four weeks postoperatively (middle panel), and six months postoperatively (lower panel). The color coding, axes, and lines are the same as in Figure 3. No mean sweat pattern curve is shown for patients with palmar hyperhidrosis due to the small sample size.

(A)

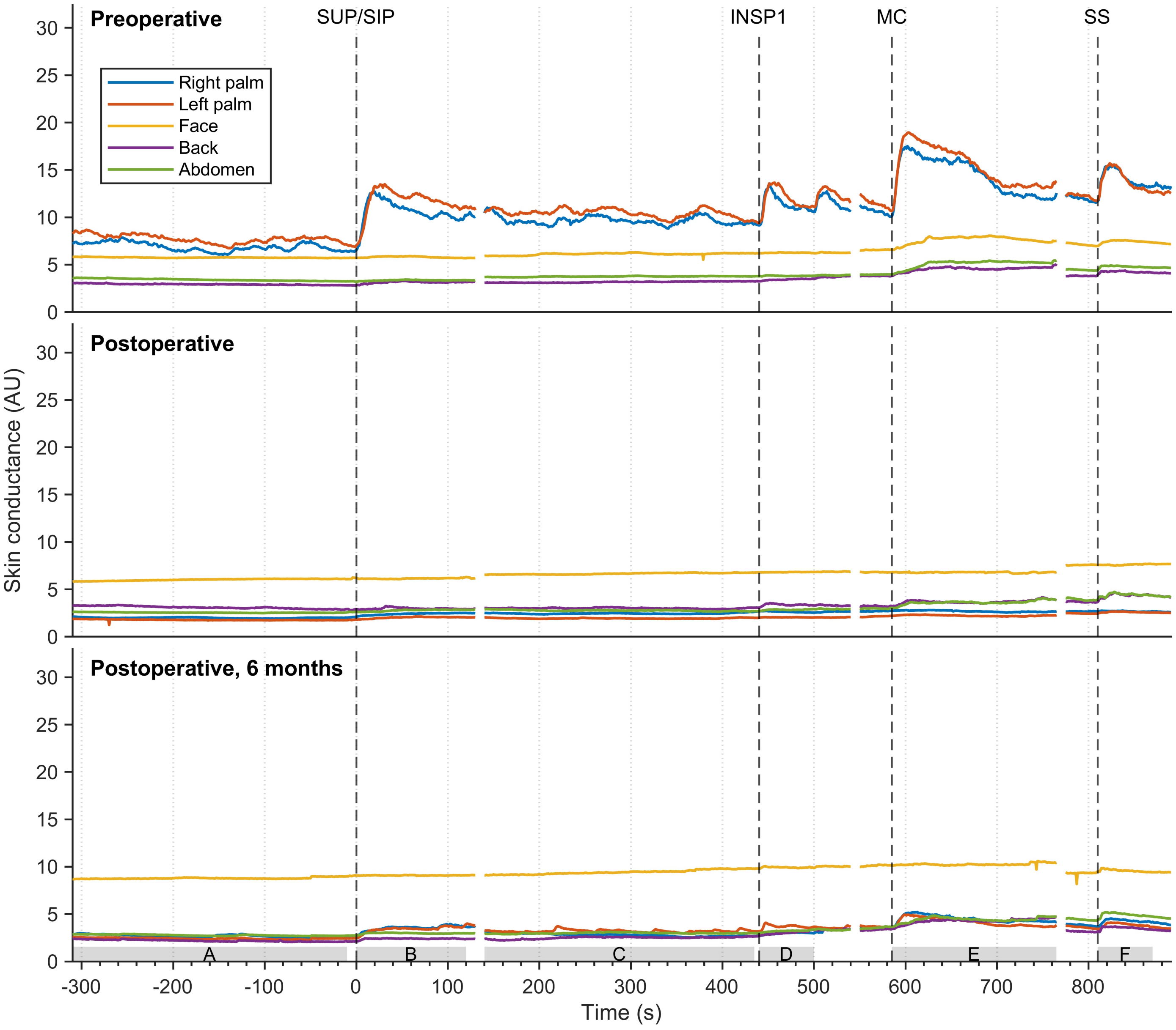
Preoperative
Postoperative
Postoperative, 6 months
SUP/SIP
INSP1
MC
SS
Right palm
Left palm
Face
Back
Abdomen
Skin conductance (AU)
Time (s)
A
B
C
D
E
F
-300
-200
-100
0
100
200
300
400
500
600
700
800
0
5
10
15
20
25
30

(B)

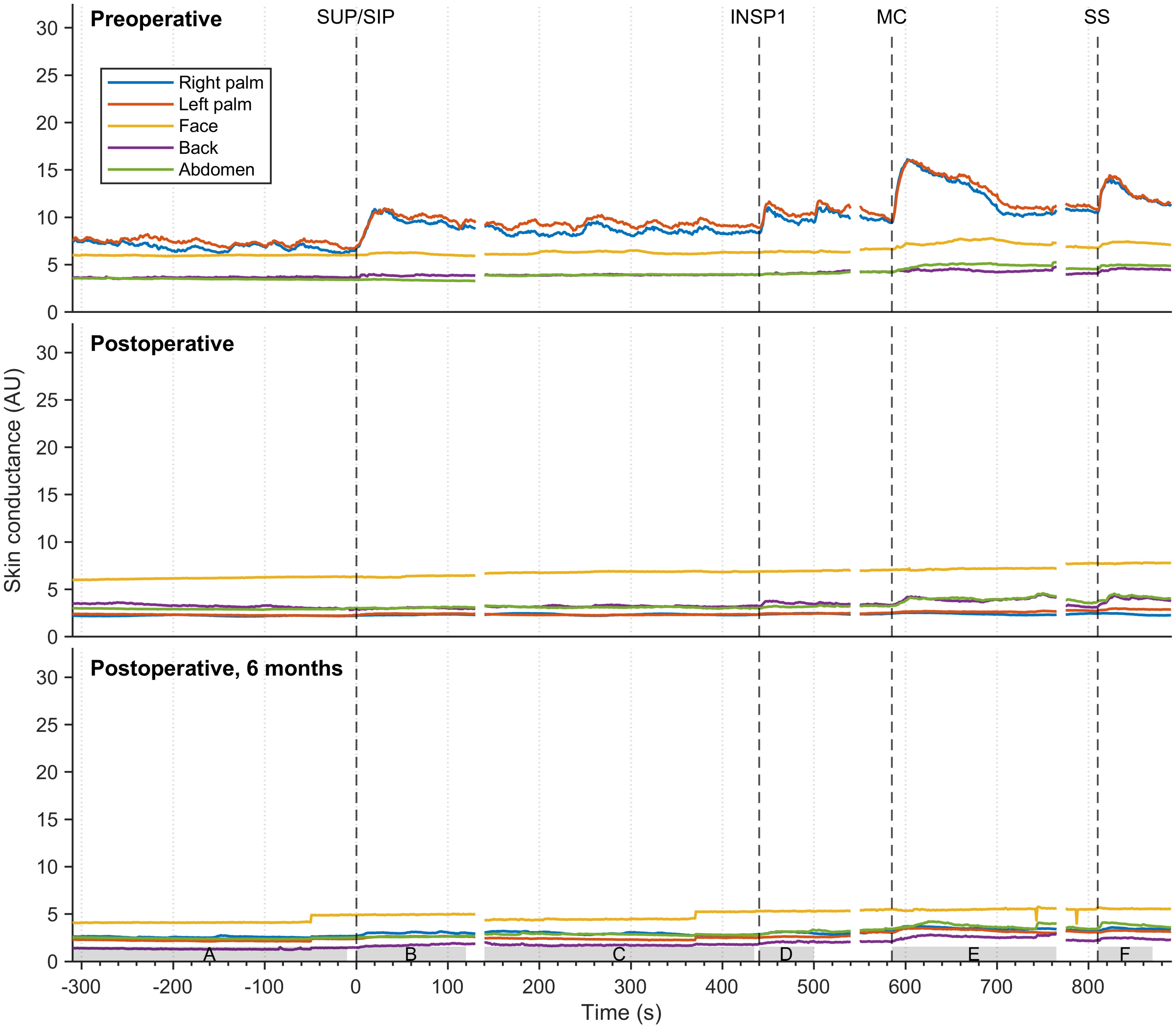

Preoperative
Postoperative
Postoperative, 6 months
SUP/SIP
INSP1
MC
SS
Right palm
Left palm
Face
Back
Abdomen
Skin conductance (AU)
Time (s)
A
B
C
D
E
F

(C)

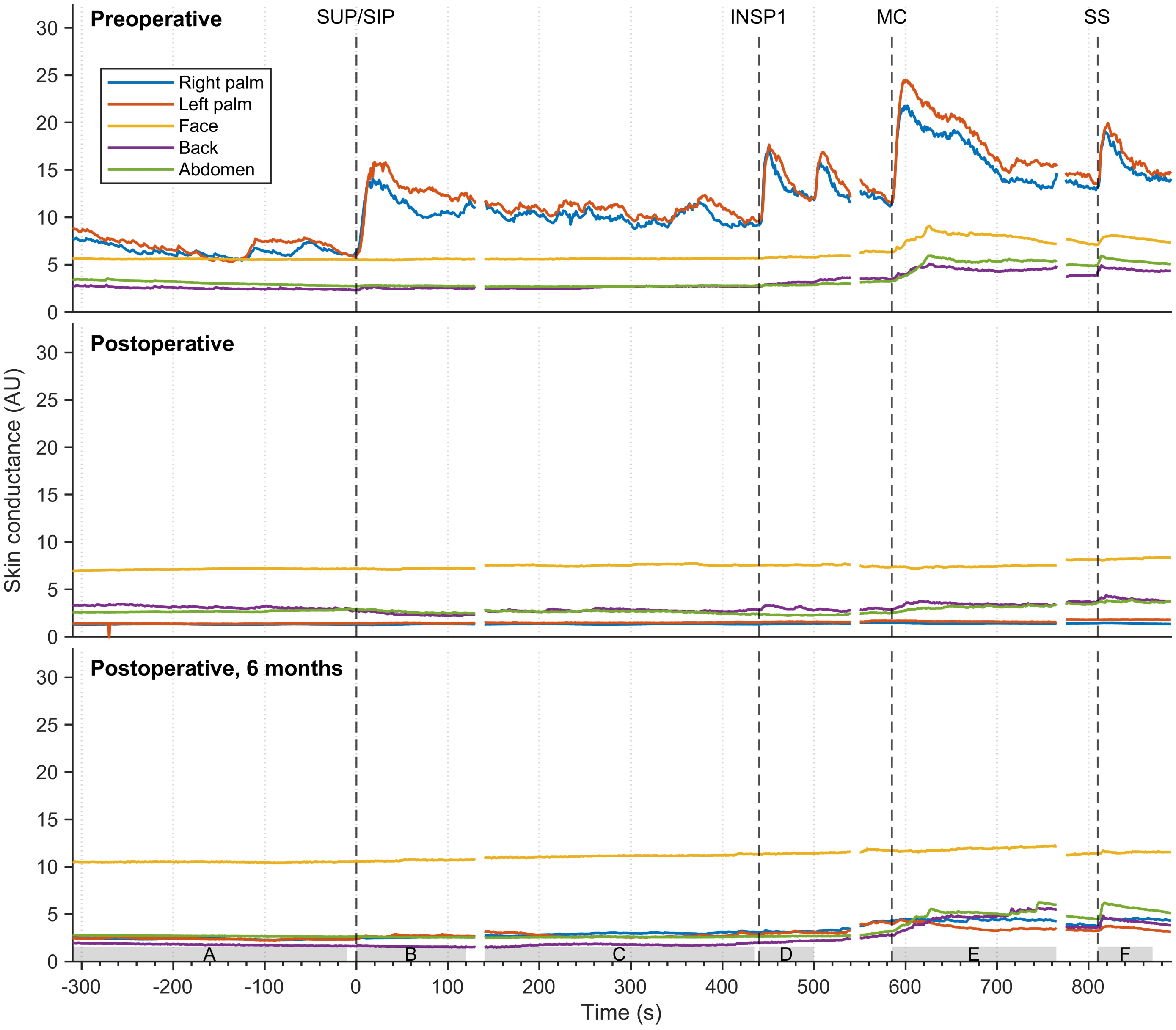

Preoperative
Postoperative
Postoperative, 6 months
SUP/SIP
INSP1
MC
SS
Right palm
Left palm
Face
Back
Abdomen
Skin conductance (AU)
Time (s)
A
B
C
D
E
F
